# Cyanoremediation of heavy metals (As(v), Cd(ii), Cr(vi), Pb(ii)) by live cyanobacteria (*Anabaena variabilis*, and *Synechocystis* sp.): an eco-sustainable technology†

**DOI:** 10.1039/d4ra00409d

**Published:** 2024-04-02

**Authors:** Md. Sabbir Hossain, Tatsufumi Okino

**Affiliations:** a Graduate School of Environmental Science, Hokkaido University Sapporo 060-0810 Japan okino@ees.hokudai.ac.jp; b Department of Environmental Science and Technology, Jashore University of Science and Technology Jashore-7408 Bangladesh; c Faculty of Environmental Earth Science, Hokkaido University Sapporo 060-0810 Japan

## Abstract

The cyanoremediation technique for heavy metal (HM) removal from wastewater using live cyanobacteria is promising to reduce the pollution risk both for the environment and human health. In this study, two widely recognized freshwater cyanobacteria, *Anabaena variabilis* and *Synechocystis* sp., were used to explore their efficacy in HM (As(v), Cd(ii), Cr(vi), Pb(ii)) removal. The different optimum adsorption conditions were pH 8 and 7.5 for *A. variabilis* and *Synechocystis* sp., respectively, but the temperature (25 °C) and contact time (48 hours) were the same for both strains. Under these specified conditions, *A. variabilis* exhibited the capability to remove 25% of As(v), 78% of Cd(ii), 54% of Cr(vi), and 17% of Pb(ii), whereas *Synechocystis* sp. removed 77% of As(v), 57% of Cd(ii), 91% of Cr(vi), and 77% of Pb(ii) at different initial concentrations. Metal diversity interfered negatively with cyanobacterial growth, especially Cd(ii) and As(v), as measured by OD_730_, dry biomass, chlorophyll a, and carotenoid production for both strains. Fourier transform infrared spectrum (FT-IR) analysis revealed the existence of diverse surface binding sites for HM adsorption, stemming from proteins and polysaccharides. Scanning Electron Microscopy (SEM) and Energy Dispersive X-ray Spectroscopy (EDS) confirmed the presence of HMs on the surface of the cyanobacterial cells. Finally, the zeta potential results indicating alterations in the surface negative charges elucidated the adsorption mechanisms involved in the HM removal by both cyanobacteria. These results provided a comprehensive understanding of the HM adsorption mechanism by cyanobacteria, offering valuable theoretical insights that can be extrapolated to enhance our comprehension of the cyanoremediation mechanisms by various other cyanobacterial strains.

## Abstract 2

Cyanoremediation of heavy metals by surface adsorption of live cyanobacteria represents a promising technique for wastewater treatment.

## Graphical abstract

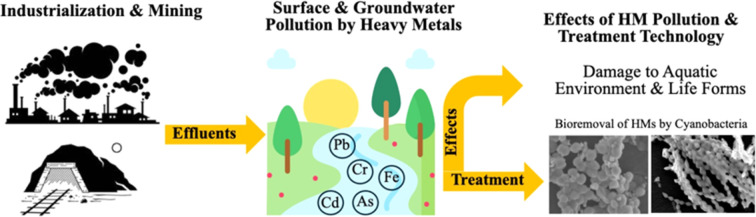

## 1. Introduction

Heavy metals (HMs) are widely recognized as one of the most preeminent environmental pollutants, owing to their intrinsic toxicity, persistence, and bio-accumulation proclivity within biota. These contaminants pose substantial ecological risks to the ecosystem and can lead to severe consequences.^1^ In a biological system, some HMs, such as Cu, Mn, Zn, and Cr, play the role of micronutrients, but when they exceed the permissible limits, they are considered contaminants and can bring physiological damage to health.^2^ The persistent HMs can be magnified within the trophic levels of the food chain, inheriting mutagenic and/or carcinogenic potential for human beings.^3^ The toxicity of HMs (As(v), Cd(ii), Cr(vi), Pb(ii)) has become a global issue because of their presence in the food chain, as human beings are the top consumers in the food chain.^4^ HMs in drinking water can cause chronic effects, including nausea, vomiting, diarrhea, kidney failure, cognitive impairment, thyroid abnormalities, muscle weakness, and cancer.^5^ Chronic exposure to Cd(ii), Cr(vi), Ni(ii), and Pb(ii) in humans may cause kidney and bone damage, adverse respiratory effects, contact allergies, and neurotoxicity, respectively.^6^ From 1972 to 2017, a discerning trend has emerged: a significant portion of the world's rivers and lakes have succumbed to contamination due to industrialization and urbanization, primarily attributed to the presence of 12 heavy metals (Al, As, Cd, Co, Cr, Cu, Fe, Hg, Mn, Ni, Pb, and Zn).^7^ The HM concentrations in surface water exhibit pronounced disparities. Surface water in developing countries of Africa, Asia, and South America demonstrates higher levels of HMs compared to the developed nations of Asia, Europe, and North America. Natural (geological weathering of bedrock) and nonanthropogenic (mining activities) sources are responsible for HM deposition in the water and sediments of rivers and lakes,^8^ and the HMs undergo various alterations in their chemical forms by dissolution, precipitation, and complexation processes while being transported from their sources.^9^ Bangladesh, India, Pakistan, China, and Nigeria are experiencing HM pollution in groundwater due to industrial discharge, agricultural pesticides, and inadequate wastewater treatment systems.^10,11^

Traditional methods for HM removal from wastewater are mainly physical adsorption, ion exchange, reverse osmosis, electrodialysis, chemical precipitation, coagulation, solvent extraction, ultrafiltration, and nanofiltration, which are expensive and generate secondary pollution causing further deterioration of water and soil properties. These limitations of conventional treatment technologies have contributed to the increased popularity of nature-based biological treatment techniques.^12^ Nature-based systems (NBSs) stand as a viable technological option for wastewater treatment, owing to their consistent, efficient, and cost-effective approaches, which aligns with the pursuit of sustainable development goals (SDGs) by emphasizing the principles of a circular economy.^13^ The biological alternatives to artificial remediation, commonly referred to as bioremediation, represent a cost-effective and environmentally friendly approach. This bioremediation method involves harnessing microorganisms (cyanobacteria, fungi, bacteria) to reduce the HM concentration.^14^

Bioremediation or bioremoval of HMs using ubiquitous autotrophic cyanobacteria is called cyanoremediation,^3^ and is being used for soil and wastewater treatment due to its cost-effective and environment-friendly nature. The small size, ability to withstand harsh environments, simple nutrient requirements, high surface-to-volume ratio, greater mucilage volume, and detoxification mechanism of cyanobacteria bestow upon them the capability of efficient bioremoval of HMs.^15,16^ The outer membrane of cyanobacteria, called the envelope, sheath, or capsule, is mainly composed of polysaccharides.^17^ The presence of numerous binding sites on the cell surface of cyanobacteria makes them suitable candidates for cyanoremediation of HMs from aqueous solution. These anionic binding sites serve as the receptor for heavy metals. The presence of negative charges on the exopolysaccharide (EPS) producing cyanobacteria is also considered promising for the removal of positively charged HMs from aqueous solution.^18,19^ Polysaccharides, proteins, lipids, and other polyelectrolytes are the primary components of the cyanobacterial cell wall, and the presence of different functional groups forms negative charges on the cell wall to form coordination complexes with metal ions.^20^

Cyanobacteria can remove HMs by active and passive processes. The active process is called bioaccumulation, which is an irreversible slower process, where metals are assimilated by cell metabolism. This cytoplasmic metal bioaccumulation happens *in vivo* as part of the cyanobacterial defense mechanism for the toxic HMs. In the passive process, metals are adsorbed onto the cell wall and by exterior functional groups, also called biosorption.^21,22^ The passive adsorption mechanism is a quick and reversible process. Still, most of the related studies have focused on the cyanobacterial efficiency of HM removal, and the adsorption mechanisms have not been emphasized and the adsorption mechanism still needs to be clarified. Also, the removal of different HMs by the same cyanobacterial species has not been well studied and there is inadequate information about the standard initial working concentration of HMs for future research in the field of cyanoremediation. Hence, this study has established the foundation for a comprehensive understanding of the adsorption mechanism of *Synechocystis* sp. and *A. variabilis* in response to four different HMs, also providing insights for other cyanobacterial species. In this study, the HM removal efficiency of cyanobacteria and the adsorption mechanism were studied. The influence of solution pH, initial metal concentration, and cell viability after a specific contamination time were also studied. The effects of HMs in terms of OD_730_, chlorophyll a, carotenoids, and dry biomass were also investigated. FT-IR, SEM, EDS, and surface zeta potential change explained the adsorption mechanism of the metals. Finally, this research tried to set a reference standard of the initial HM concentration for future wastewater treatment research by the cyanoremediation technique.

## 2. Materials and methods

### 2.1 Cyanobacteria culturing conditions

Pure cultures of *Synechocystis* sp. (NIES-3758) and *A. variabilis* (NIES-2095) were obtained from the National Institute for Environmental Studies (NIES), Japan, and cultured in BG-11 and MDM medium, respectively. Prior to cultivation, all of the experimental apparatus and culture medium were autoclaved at 121 °C for a duration of 30 min to prevent contamination. Both strains were cultured incrementally, transitioning from small scale (10 mL) to large scale (50 mL, 500 mL, and 10 L) to ensure the cultivation of robust and healthy cells. During cell culture in a 1000 mL Erlenmeyer flask, the optimum pH for *Synechocystis* sp. and *A. variabilis* was maintained at 7.5 and 8, respectively.^23^ Cultures were maintained in a controlled environment chamber under the following conditions: 25 °C, 12/12 h light/dark cycle with 2000 lx light intensity, and 60% relative humidity.^24^

### 2.2 Growth and photosynthetic pigments of cyanobacteria under HM contamination

Cyanobacterial growth was determined by measuring the optical density at 730 nm (ref. 25) (hereafter OD_730_) and dry biomass (mg L^−1^) with and without HM contamination. The initial biomasses of both cyanobacteria based on OD_730_, dry biomass, chlorophyll a, and carotenoids were the same (OD_730_ > 1.5) to ensure synchronized cultivation. For *Synechocystis* sp., OD_730_ and dry biomass were measured up to 10 days at intervals of 2 days. To assess the effects of HMs on cyanobacterial growth, the concentration of HMs selected corresponds to the point at which the maximum removal of HMs was observed. Specifically, for *Synechocystis* sp., this concentration was determined to be 1 mg L^−1^ for As(v), 1 mg L^−1^ for Cd(ii), 2 mg L^−1^ for Cr(vi), and 4 mg L^−1^ for Pb(ii); and for *A. variabilis*, the corresponding concentrations were 4 mg L^−1^ for As(v), 1 mg L^−1^ for Cd(ii), 1 mg L^−1^ for Cr(vi), and 1 mg L^−1^ for Pb(ii). OD_730_ was recorded using an ultraviolet spectrophotometer (Jasco V-650 spectrophotometer). Chlorophyll a and carotenoids were measured by following the previously reported method,^26^ employing formulae (1) and (2) under specific conditions (Table S1†). All experiments were performed in triplicate.

1 Chl_a_ [μg ml^−1^] = 12.9447(*A*_665_ − *A*_720_)

2 Carotenoids [μg ml^−1^] = [1000(*A*_470_ − *A*_720_) − 2.86(Chl_a_ [μg ml^−1^])]/221

### 2.3 Cell viability

Cell viability was counted according to the previously reported method^27^ using trypan blue dye on a BIO-RAD TC10™ automated cell counter. A 20 μL aliquoted cell suspension was mixed with 20 μL of trypan blue dye in the dual chambers of counting slides. After inserting the counting slide into the BIO-RAD, total cell count, live cell count, and percentage of live cells were automatically measured within a span of 30 s and this procedure was performed in the sterile environment of a clean bench. All sample tests were performed in three biological replicates.

### 2.4 HM stock solution preparation

The aqueous stock solution of As(v), Cd(ii), Cr(vi), and Pb(ii) ions was prepared by dissolving NaAsO_2_, CdCl_2_, KCr_2_O_7_, and PbNO_3_ in distilled water, respectively, with a concentration of 1000 mg L^−1^. From this stock solution, various experimental concentrations were prepared in a 1 L Erlenmeyer flask by diluting this stock solution with sterilized BG11 and MDM medium. The final concentrations were as follows: for As(v) and Cr(vi) – 1, 2, 3, 4, 5, and 10 mg L^−1^; for Pb(ii) – 1, 2, 4, 5, 6, and 8 mg L^−1^; and for Cd(ii) – 0.2, 0.4,0.5, 0.6, 0.8, 1, 1.5, and 2 mg L^−1^. Depending on the metal toxicity, we considered various initial working concentrations. Cells cultured without HMs were considered as controls. Three replicates were used for each concentration treatment. All the chemicals used were analytical grade and purchased from Sigma-Aldrich.

### 2.5 Quantification of the HM concentrations adsorbed by cyanobacteria

To investigate the role of *Synechocystis* sp. and *A. variabilis* for HM removal, a series of experiments were conducted. A 100 mL aliquot of cyanobacterial cell culture was transferred to an Erlenmeyer flask during their log phase for contamination with HMs of different concentrations. The initial OD7_30_ was >0.45 to provide sufficient biomass for metal uptake analysis. The cultures in the incubator were gently shaken twice a day (in the morning and the evening) manually. After 48 hours of culture at 25 °C temperature, with a 12/12 h light/dark cycle with 2000 lx light intensity, and at 60% relative humidity in a culture room, 5 mL of cells were centrifuged at 3000 rpm for 15 min to separate them from the medium and the cell pellets were acid digested.^28^ The cell pellets were placed in a 5 mL mixture of HNO_3_ and HClO_4_ (2 : 1, v/v) and digested at 121 °C until transparent. The resulting solution was diluted to 10 mL with deionized water and filtered through a 0.22 μm cellulose acetate membrane filter, and the HMs in the cells were analyzed by Inductively Coupled Plasma Mass Spectroscopy (ICP-MS, Agilent Technologies G3663A) and Atomic Absorption Spectrometry (HITACHI 180-30 A.A.S.). The HM removal efficiency was calculated using the following formula:
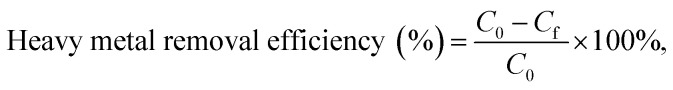
where *C*_0_ and *C*_f_ are the initial and residual concentration of HMs, respectively. Three experiments were conducted to ensure the accuracy of the results. ICP-MS, within the spectrum of analytical methodologies, is distinguishable by its ability to circumvent the necessity for intricate and time-consuming sample pre-processing protocols. Additionally, it excels in terms of its resiliency concerning delivering high levels of precision, accuracy, and selectivity.^29^

### 2.6 Active center surface analysis by Fourier-transform infrared (FTIR)

The presence of discernible functional groups on the surface of cyanobacteria was assessed by Fourier transform infrared spectroscopy (FT-IR) (Thermo Scientific, Nicolet iS10). HM accumulated cells were separated by centrifugation at 3000 rpm for 15 min from the medium. The cell pellets were washed twice with deionized water and lyophilized by a freeze-dryer. The dried sample was ground into a powder using a mortar and pestle. A sample of 1 mg was ground with 100 mg of potassium bromide (KBr) powder (1 : 100) and compressed into a thin disc of 3 mm diameter.^30^ The initial HM concentrations for the FT-IR samples are mentioned in the supporting data, Table S1.† The FT-IR spectrum was then obtained in transmittance mode to analyze various functional groups within the frequency range of 4000–400 cm^−1^.

### 2.7 SEM and EDS analysis

Morphological changes in the cell surface can be identified by scanning electron microscope (SEM) observation. To corroborate the surface attachment of HMs to the cyanobacterial cells, SEM (JEOL, JSM-6510LA) and EDS (energy-dispersive X-ray spectroscopy) (JEOL, JED-2300) analysis were conducted between 2000–5000× magnification. The image was acquired by a secondary electron detector with an accelerating voltage of the electrons of 10–20 kV. After HM adsorption at specific concentrations (Table S1†), the cyanobacterial cell surface morphology was analyzed by SEM analysis and compared with the surface morphology of the cells without HM adsorption. To obtain an SEM image, the cultures were harvested and lyophilized, and then the dried cells were coated (JEOL JFC-1600, AUTO FINE COATER) with a thin layer of platinum and examined using SEM-EDS. Quantitative analysis and the elemental percentage of HMs were determined by EDS.

### 2.8 Zeta potential

The zeta potential of *A. variabilis* and *Synechocystis* sp. was assessed both before and after HM adsorption, allowing for a comparison of the changes in net cell surface charge. The zeta potential was measured following the method described in previous research.^31^ Cyanobacterial cells (0.01 g) were suspended in a 0.01 M KCl solution and stirred for 10 min to ensure thorough mixing. Subsequently, the pH was adjusted (Table S1†) using 1 M of NaOH or HCl. The solution was left undisturbed for 15 min, and the supernatant was collected for zeta potential measurement utilizing a zeta potential analyzer (Zetasizer Nano ZS90, Malvern). To ensure precision and reproducibility, three independent zeta potential measurements were conducted for each sample. All experiments were performed in triplicate.

## 3. Results and discussion

### 3.1 HM removal ability of cyanobacteria

The metal uptake potential of *Synechocystis* sp. exhibited a decline with an increase in the initial concentration of the added metal.

**Fig. 1:**
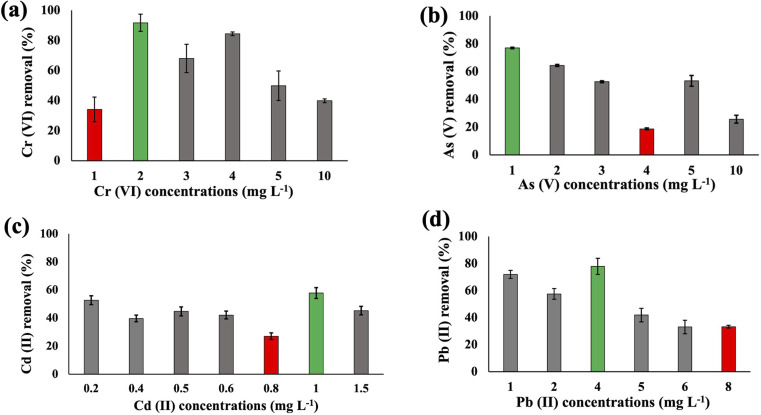
The heavy metal Cr(vi) (a), As(v) (b), Cd(ii) (c), and Pb(ii) (d) removal efficiency of *Synechocystis* sp. after 48 h at pH 7.5 with different initial concentrations. Error bars indicate the standard deviation. Green and red bars indicate the maximum and minimum removal efficiency, respectively.

The overall metal uptake order across all experimental sets appeared as Cr(vi) > As(v) > Cd(ii) > Pb(ii). From Fig. 1, it is evident that for *Synechocystis* sp. with increasing concentration of HMs, the removal efficiency decreased, which is also in agreement with the research from Shen *et al.*^32^*Synechocystis* sp. exhibited significant HM removal efficiency, including 91% for Cr(vi) at 2 mg L^−1^ initial concentration, 77% for As(v) at 1 mg L^−1^ initial concentration, 57% for Cd(ii) at 1 mg L^−1^ initial concentration, and 77% for Pb(ii) at 4 mg L^−1^ initial concentration (Fig. 1). Among the four HMs, *Synechocystis* sp. demonstrated the highest adsorption percentage for Cr compared to the other metals, indicating a greater affinity of this cyanobacterium for Cr(vi) adsorption. This observation aligns with a similar finding reported in previous research by Khattar *et al.*^33^ Wang *et al.*^34^ also reported a similar result that Cd(ii) and Cr(vi) adsorption by *Synechocystis* sp. PCC6803 decreased at higher initial concentration and the predominant mechanism was extracellular adsorption.

**Fig. 2:**
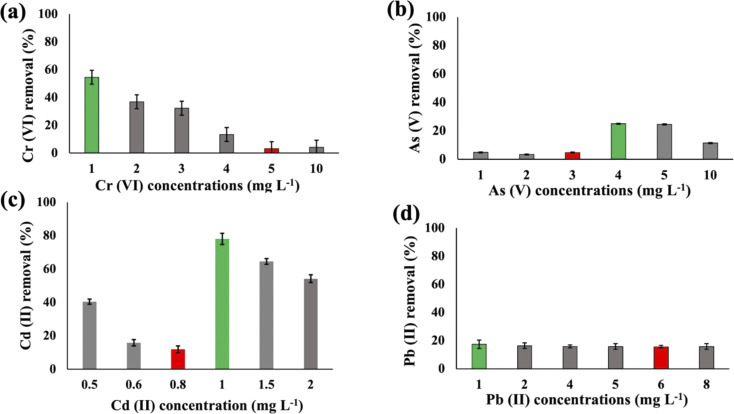
Heavy metal (Cr(vi) (a), As(v) (b), Cd(ii) (c), and Pb(ii) (d)) removal efficiency of *A. variabilis* after 48 h at pH 8 with different initial concentrations. Error bars indicate standard deviation. Green and red bars indicate the maximum and minimum removal efficiency, respectively.

Substantial variations in the metal absorption values by *A. variabilis* were observed across distinct initial metal concentrations. The optimum metal removal efficiency of *A. variabilis* varies across different concentrations for each metal (Fig. 2). The efficiency of *A. variabilis* in removing Cr(vi) ions was more pronounced at lower initial concentrations. This examined cyanobacteria could remove 54% of Cr(vi) from the wastewater at 1 mg L^−1^ initial concentration and the removal efficiency decreased with increasing concentration. At higher concentrations of 5 mg L^−1^ and 10 mg L^−1^, the removal efficiency was negligible. *A. variabilis* could remove 25% of As(v) at 4 mg L^−1^ initial concentration and at both lower and higher concentrations the removal efficiency was nearly negligible. When the initial concentration of Cd(ii) was 1 mg L^−1^, the removal efficiency was 78%, while for the same concentration of Pb(ii), the removal efficiency was only 17%. *A. variabilis* showed the highest Cd(ii) removal efficiency at 1 mg L^−1^ and this capacity started to decrease with increasing concentration. Abdel-Aty *et al.*^35^ reported a parallel outcome, indicating that the efficiency of *Anabaena sphaerica* in removing Cd(ii) and Pb(ii) decreased with an increase in the initial concentration of these HMs. These results establish the potential of two cyanobacterial strains *A. variabilis* and *Synechocystis* sp. biomass as a viable biosorbent for the removal of Cd(ii) and Pb(ii) from aqueous solutions. In this single metal system, the electronegativity order is Pb(ii) > As(v) > Cd(ii) > Cr(vi), indicating that there is no direct correlation between the electronegativity of the HMs and the sorbent affinity of both cyanobacteria. Instead, the trends in HM removal efficiency depend on the toxicity of the metals and their concentrations. *Synechocystis* sp. typically forms unicellular or small multicellular colonies, whereas *A. variabilis* forms long filamentous structures. Additionally, *A. variabilis* possesses specialized cells called heterocysts, which are absent in *Synechocystis* sp., enabling *A. variabilis* to perform nitrogen fixation more efficiently. These morphological characteristics influence their cyanoremediation potential for HMs.

Higher concentrations of HMs can be lethal to the cyanobacteria, but different species may exhibit distinct trends in their susceptibility to lethality in response to varying concentrations of metals. Despite the prevalence of dead cells in the wastewater culture, it is crucial to acknowledge that the biosorption process can take place on the surfaces of both dead and live cyanobacterial cells. This phenomenon contributes to the removal of heavy metals from wastewater.^36^

Optimal pH conditions were employed to facilitate the maximum growth of the cyanobacteria. *Synechocystis* sp. and *A. variabilis* were cultured at pH 7.5 and 8.0, respectively, which represent slightly basic conditions necessary to maintain an appropriate medium environment for their optimal growth. This pH range was chosen to ensure the well-being of the cyanobacteria, as they can be adversely affected by extreme acidic or basic conditions. Furthermore, the cyanobacteria were cultured at a fixed pH as both high and low pH can exert a substantial impact on HM adsorption. In low pH solutions (pH < 2.5), an abundance of H^+^ ions compete with the HMs on the cell surface, generating a repulsive force between the HMs and the binding sites during the adsorption process. Conversely, at high pH values, metal ions can precipitate from the alkaline solutions, resulting in a decrease in adsorption efficiency.^37^

### 3.2 Effects of HMs on cyanobacterial growth

**Fig. 3:**
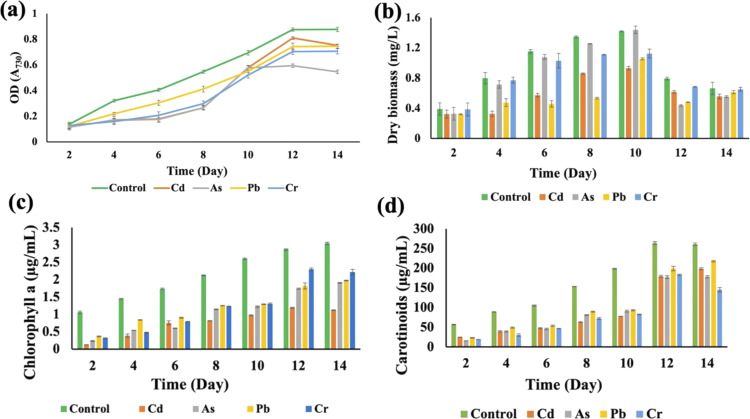
Effects of HMs on optical density (OD_730_) (a), dry biomass (b), chlorophyll a (c), and carotenoid (d) production of *A. variabilis* under HM (1 mg L^−1^ Cd(ii), 4 mg L^−1^ As(v), 1 mg L^−1^ Pb(ii), and 1 mg L^−1^ Cr(vi)) stress during the cultivation time. Error bars indicate standard deviation.

*Synechocystis* sp. and *A. variabilis* were exposed to the concentrations of metals at which they exhibited the maximum HM removal efficiency to investigate the impact of HMs on their growth. Growth of cyanobacteria was monitored for a longer period (Table S1†) during HM adsorption and compared with the control culture, which was free of HM contamination. The results showed the interference of the diversity of metals in cyanobacterial growth. OD_730_, dry biomass weight, chlorophyll a, and carotenoid production showed clear results that HMs affect cyanobacterial growth and cell viability. The OD_730_ for *A. variabilis* showed that after 12 days, it started to decline. Although Pb(ii), Cr(vi), and Cd(ii) have a similar trend, As(v) has the most negative effect on this strain (Fig. 3(a)). The dry biomass of *A. variabilis* also showed the negative effects impeded by HMs. It was observed that the control culture exhibited a consistent growth of cyanobacterial biomass, whereas the experiment with HM contamination exhibited a diminished growth in biomass. The dry biomass of *A. variabilis* started to decline after 10 days and on the 12^th^ day, As(v) showed the most negative effect on this strain (Fig. 3(b)). Chlorophyll and carotenoid production increased sharply for the control group until the 14^th^ day. During this growth period, Cd(ii) and As(v) showed the most negative effect on this strain in terms of chlorophyll and carotenoid production compared to the control group (Fig. 3(c) and (d)). This may be attributed to the ease with which Cd(ii) and As(v) can damage the protein structures.^31^ El-Hameed *et al.*^38^ used cyanobacteria *T. variabilis* and *N. muscorum* and showed that these two cyanobacteria under Cd(ii) contamination exhibited a reduction in chlorophyll and carotenoid production after 14 days.

**Fig. 4:**
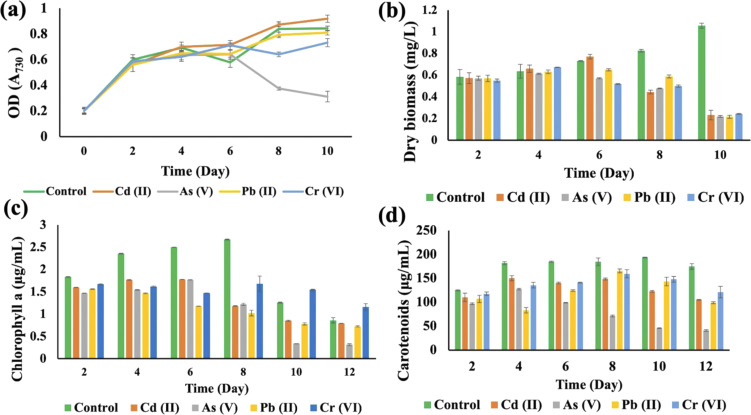
Effects of HMs on optical density (OD_730_) (a), dry biomass (b), chlorophyll a (c), and carotenoids (d) of *Synechocystis* sp. under different HM (1 mg L^−1^ Cd(ii), 1 mg L^−1^ As(v), 4 mg L^−1^ Pb(ii), and 2 mg L^−1^ Cr(vi)) stress during the cultivation time. Error bars indicate the standard deviation.

The growth curves of *Anabaena variabilis* and *Synechocystis* sp. without HMs, measured in terms of OD_730_, demonstrated consistency (Fig. 3(a) and 4(a)). The growth of *Anabaena variabilis* exhibited an increase until the 12th day, followed by a decline starting from the 14th day. Conversely, the OD_730_ of *Synechocystis* sp. peaked on the 10^th^ day before gradually declining. Based on this growth curve analysis, it is evident that the growth of *A. variabilis* surpasses that of *Synechocystis* sp. to some extent.

HMs interfered with the growth of *Synechocystis* sp., affecting OD_730_, dry biomass, chlorophyll a, and carotenoid production when compared to the control group (Fig. 4). The OD_730_ result was recorded for 10 days and it was found that As(v) had the most negative impact, while it started decreasing after 10 days. Pb(ii) gave results that were similar to the control group (Fig. 4(a)). For the dry biomass production, As also showed the most pronounced negative impact on this strain (Fig. 4(b)). Chlorophyll production increased sharply until the 8^th^ day for the control group, but on the 10^th^ day As(v) contaminated *Synechocystis* sp. produced less chlorophyll a compared to other HM contaminated strains (Fig. 4(c)). Carotenoid production increased until 10 days, but the As(v) contaminated strain experienced the most negative impact in terms of carotenoid production (Fig. 4(d)). From the 8^th^ day, Cr(vi) contaminated *Synechocystis* sp. demonstrated a noteworthy increase in chlorophyll and carotenoid production compared to the control group. For both *Synechocystis* sp. and *A. variabilis*, exposure to As(v) contamination resulted in the most adverse impacts on dry biomass, chlorophyll a, and carotenoid production when compared to other HMs. Some essential metals bind to specific proteins, and it is a big challenge for the cells to transfer the specific metal to specific proteins. These metalloproteins in cyanobacterial cells are responsible for metal binding with the help of enzymes, but when the metal concentration increases in the cells, it causes toxic effects in cells, which finally affects cell growth. As the metalloproteins follow an order for metal binding and particular metalloproteins are responsible for specific metal binding, toxicity varies from metal to metal.^39^ In this research, we have also found that none of the metal species produce similar growth effects.

### 3.3 Cell viability

**Fig. 5:**
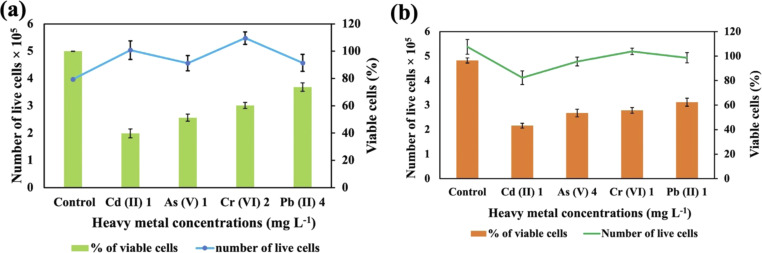
Cell viability counting of *Synechocystis* sp. (a) and *A. variabilis* (b) after 48 h of HM adsorption. Data are expressed as means ± standard errors (*n* = 3).

These two strains were subjected to the concentrations at which they exhibited the maximum removal efficiency of HMs to ascertain cell viability. It was observed that *Synechocystis* sp. demonstrated survival rates of 40%, 51%, 60%, and 74% at concentrations of 1 mg L^−1^ for Cd(ii), 1 mg L^−1^ for As(v), 2 mg L^−1^ for Cr(vi), and 4 mg L^−1^ for Pb(ii), respectively (Fig. 5(a)). *A. variabilis* could maintain survival rates of 54%, 43%, 56%, and 62%, at concentrations of 4 mg L^−1^ for As(v), 1 mg L^−1^ for Cd(ii), 1 mg L^−1^ for Cr(vi), and 1 mg L^−1^ for Pb(ii), respectively (Fig. 5(b)). Notably, for both strains, only Cd(ii) exhibited an impact exceeding 50% on cell viability, while the other HMs caused less than 50% reduction in cell viability.

Increased concentrations of HMs were observed to be detrimental to cyanobacteria, resulting in a negative influence on cell viability. Ultimately, this toxicity resulted in the demise of the cells in the solution. The lethal concentration of HMs is contingent upon the specific cyanobacterial strains and the type of HMs. Previous research also shows that different strains exhibit varying lethal concentrations for different HMs.^36^ The resistance to As in *Synechocystis* can be conferred through an operon comprising a trio of genes: *acr3* (slr0944), *arsH* (slr0945), and *arsC* (slr0946), all of which are under the regulatory control of ArsR.^40^

### 3.4 Active center surface analysis

**Fig. 6:**
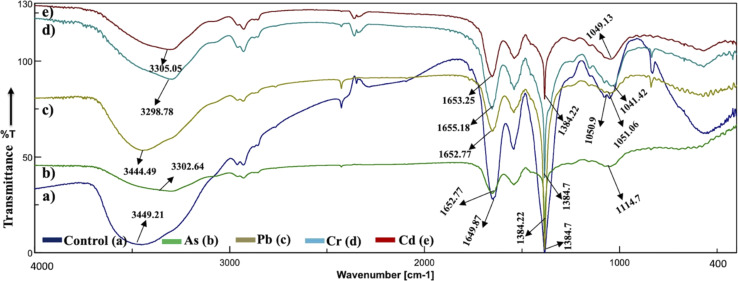
Active center analysis by FT-IR spectrum of *Synechocystis* sp. before (control – (a)) and after (As(v) – (b), Pb(ii) – (c), Cr(vi) – (d), and Cd(ii) – (e)) HM adsorption.

**Fig. 7:**
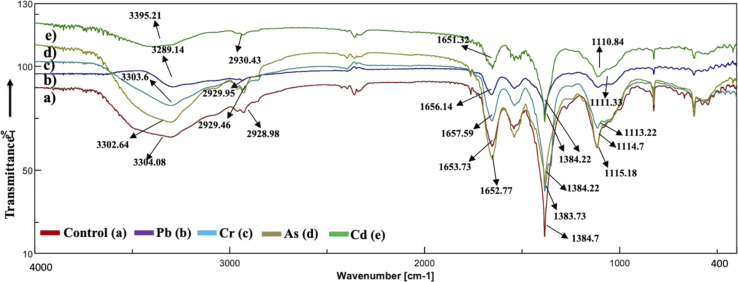
Active center analysis by FT-IR spectrum of *A. variabilis* before (Control – (a)) and after (Pb(ii) – (b), Cr(vi) – (c), As(v) – (d), and Cd(ii) – (e)) HM adsorption.

The analysis of the FT-IR spectra hinges on associating absorption bands with established absorption wavenumbers corresponding to distinct types of chemical bonds.^41^ The detected peaks falling within the 3400–3200 cm^−1^ range are ascribed to the stretching vibrations of O–H groups for the functional groups of alcohol, carboxyl, and phenol, and the vibration of N–H bonds in amines or indicative of the existence of hydrogen bonds within polymeric compounds.^42^ Although both the control and heavy metal-stressed strains exhibited nearly the same trends in the variation of their spectral peaks, there were discernible distinctions in the peak patterns for both *A. variabilis* and *Synechocystis* sp. strains. The predominant functional groups identified in the FT-IR spectra of *A. variabilis* in Fig. 7 are as follows: 3289.14–3304.8 (–OH in glucose moieties of polymeric compounds and N–H of proteins), 2928.98–2930.43 (stretching vibration of CH_2_), 1651.32–1657.59 (–CONH– a group of amide I in proteins),^43^ a shift in the peaks from 1383.73 to 1384.22 indicating deformation of C–H functional groups and –COOH,^44^ and 1110.84–1115.18 (stretching vibration of C–O–C of esters).^45^ The absorbance bands falling in the range of 1700–1500 cm^−1^ are typically linked to proteins, while those in the range of 1300–900 cm^−1^ are associated with polysaccharides (Fig. 6).^46^

In *Synechocystis* sp. (Fig. 6) the major bands shifted in the range of 3298.78–3449.21 cm^−1^, 2928.98–2930.91 cm^−1^, 1649.87–1655.18 cm^−1^, and 1041.42–1114.7 cm^−1^, which indicates stretching vibration of OH from polymeric compounds, stretching vibration of C=O and C–N (amide I) from proteins (peptidic bond), and stretching vibration of OH from polysaccharides, respectively.^16^

The spectroscopic data derived from the cyanobacterium *Synechocystis* sp. also displayed absorbance bands linked to proteins within the 1700–1500 cm^−1^ range and to polysaccharides within the 1300–900 cm^−1^ range, thereby supplying supplementary confirmation of the presence of these proteins and polysaccharide components.^44^ The peak detected within the spectral range of 1700–1600 cm^−1^ corresponds to the amide I band, indicative of the vibration of C=O bonds within the peptide groups of proteins and the peak intensities exhibit a shift after HM adsorption in comparison to the control (without HMs), indicating interactions with the C=O bond.^47^ The FT-IR results of the two cyanobacterial strains *A. variabilis*, and *Synechocystis* sp. reveal that functional groups arising from protein and polysaccharide constituents serve as pivotal participants in the binding mechanism of HMs to these thriving cyanobacterial cells. Analogous absorption band patterns and results in cyanobacterial EPS have been observed under metal-challenged conditions.^28^ Functional groups bearing negative charges undergo deprotonation at high pH levels, such as pH > 8.0. This deprotonation process amplifies the electrostatic attraction between these negatively charged functional groups and positively charged HM ions, thus augmenting the biosorption capacity,^48^ which also supports our pH range selection efficiently for HM removal by cyanobacteria.

### 3.5 Analysis of surface morphology change

**Fig. 8:**
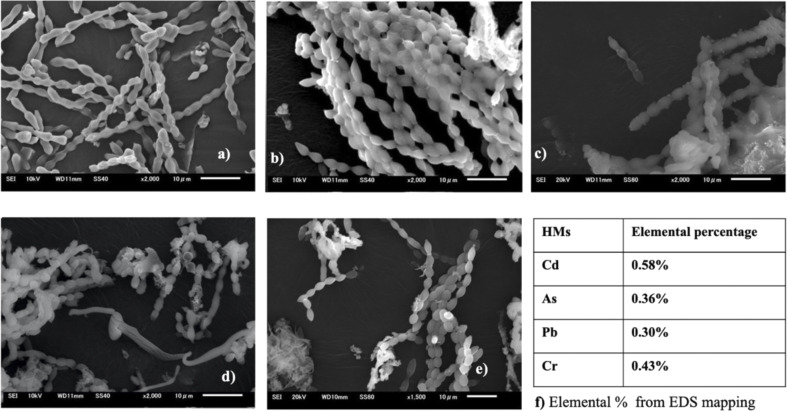
SEM images of *A. variabilis* before (a) and after Cd(ii) (b), As(v) (c), Pb(ii) (d), and Cr(vi) (e) adsorption. Quantitative analysis (f) of HMs from EDS analysis.

**Fig. 9:**
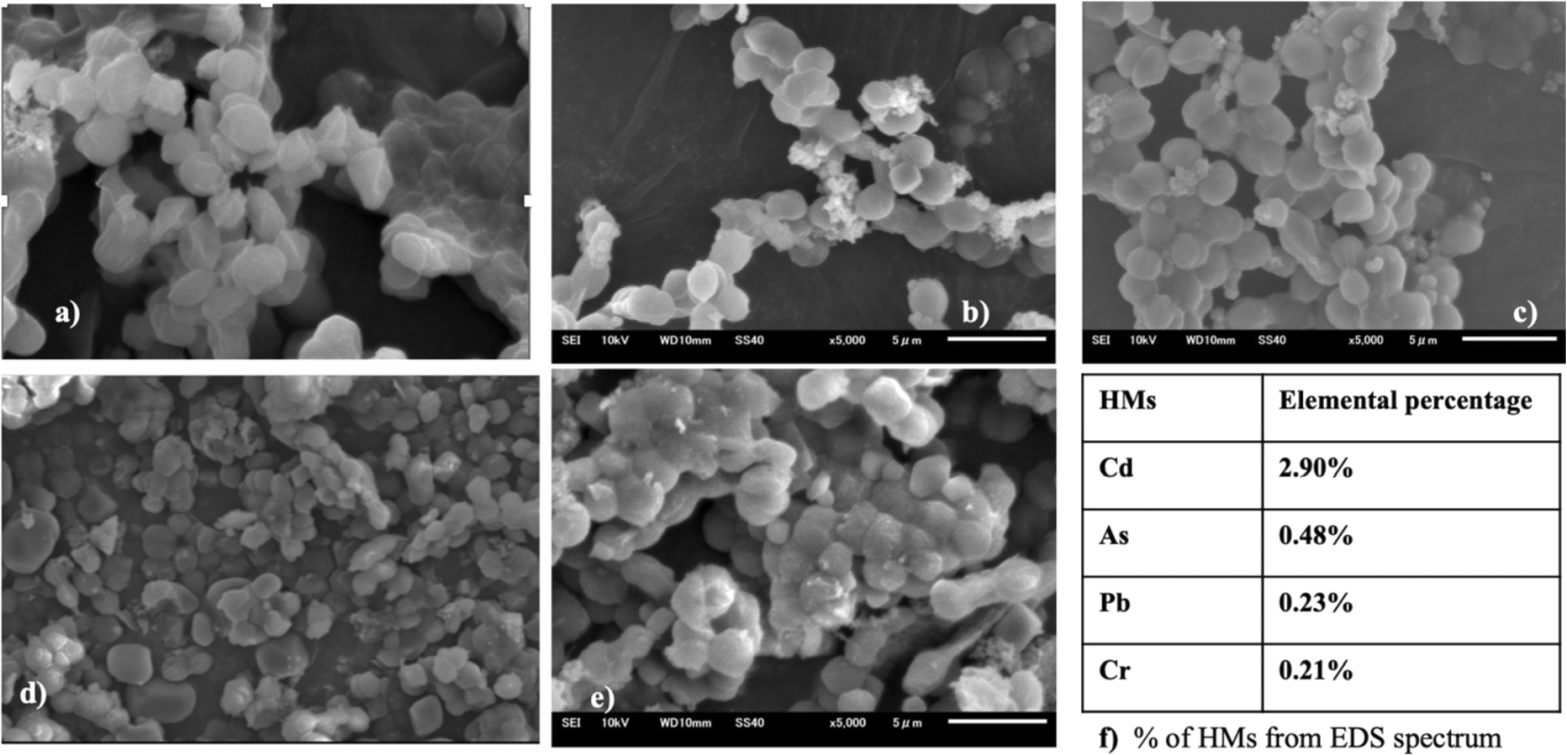
SEM images of *Synechocystis* sp. before (a) and after Cd(ii) (b), As(v) (c), Pb(ii) (d), and Cr(vi) (e) adsorption. Quantitative analysis (f) of the HMs from EDS analysis.

The interplay between metals and cyanobacteria is markedly affected by the characteristics of the cell surface. To substantiate both the surface adhesion and internalization of HMs by cyanobacterial cells and gain insight into the morphology of the cyanobacterial surface, SEM analysis was performed. The SEM images (Fig. 8 and 9) give a visual representation of *A. variabilis* and *Synechocystis* sp. cells before (a) and after Cd(ii) (b), As(v) (c), Pb(ii) (d), and Cr(vi) (e) HM stimulation. The SEM image of *A. variabilis* in Fig. 8 exposed to different HMs displayed variations in morphology, as indicated by alterations in filament length, the dimension of individual vegetative cells, and the appearance of exopolysaccharidic precipitates. The surface of *A. variabilis* was smooth before HM adsorption. The cell size exhibited a more significant change after the adsorption of Cd(ii) (Fig. 8(b)), while cell damage was predominantly observed after Pb(ii) adsorption (Fig. 8(d)) compared to the control cells (Fig. 8(a)). *A. variabilis* exhibited a higher percentage of Cd(ii) removal in comparison to other metals. This finding also provides support for the observed cell size deformation in this cyanobacterial strain. The results obtained by Singh *et al.*^49^ likewise noted a smooth surface of *Anabaena* sp. prior to metal adsorption, which is in concurrence with the findings of the current study. The EDS spectrum (Fig. S5–S9†) showed the qualitative and quantitative existence of HMs in the following order: Cd(ii) (0.58%) > Cr(vi) (0.43%) > As(v) (0.36%) > Pb(ii) (0.43%) for *A. variabilis* (Fig. 8). The SEM image (Fig. 9) of the *Synechocystis* sp. cells exhibited a haphazard crystal-coated appearance. In Fig. 9(c), the most random crystal formation was observed for As(v) adsorption. This random crystal or EPS secretion played an important role in the HM removal from the solution and promoted microprecipitation of metals.^31^ Similar results found by Naveed *et al.*^28^ reported that EPS secretion from *Synechocystis* sp. enhances the adsorption of As through a complexation mechanism. The EDS spectrum (Fig. S1–S4†) also quantifies the elemental% of the HMs in the order of Cd(ii) > As(v) > Pb(ii) > Cr(vi) (Fig. 9(f)).

Exposure to Cd(ii) can induce the synthesis of polysaccharides and EPS proteins. These proteins play a pivotal role in promoting the creation of protein coronas, consequently amplifying the adsorption of Cd(ii). Glucose and mannose represent sugar moieties that act as specific binding sites for the complexation of metals with EPS.^46,50^

### 3.6 Zeta potential of cyanobacteria

**Fig. 10:**
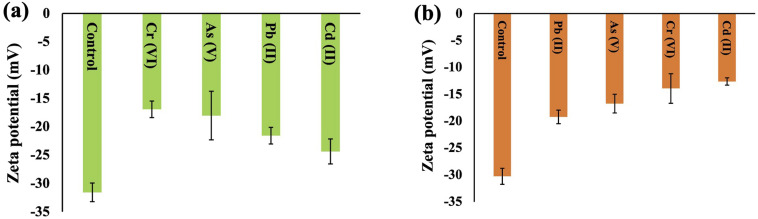
Surface zeta potential changes of *Synechocystis* sp. (a) and *A. variabilis* (b) before and after different HM adsorption. Data are expressed as means ± standard errors (*n* = 3).

Zeta potential is a parameter used to assess the stability of a colloidal or dispersion system and offers valuable insights into the net charge properties of a cell surface. The zeta potentials of *Synechocystis* sp. and *A. variabilis* were measured and a negative value was recorded for each experiment. This negative charge on the surface of the cyanobacterial cells is derived from the existence of acidic compounds, including amines, acidic polysaccharides, and carboxylic acids, which signifies the cyanobacterial cells' ability to adsorb cationic metals onto their surfaces. The zeta potential of the control cells (before HMs contamination) has a higher negativity because of the presence of negative ions, and after HM adsorption, the negativity decreases, which validates the mechanism of HM adsorption onto the cyanobacterial cell surface. The order of negativity for *Synechocystis* sp. and *A. variabilis* is control > Cd(ii) > Pb(ii) >As(v) > Cr(vi), and control > Pb(ii) > As(v) > Cr(vi) > Cd(ii), respectively (Fig. 10). In both cyanobacterial strains, the absolute zeta potential value (Tables S2 and S3†) or negativity decreases upon HM adsorption. The negativity decreases because the anionic cell surface adsorbed cationic HMs. The research conducted by Fang *et al.*^51^ similarly observed a substantial increase in Cd and Cu binding to the cell surface in conjunction with an alteration in the zeta potential values. The order of zeta potential values also indicates that *A*. *variabilis* and *Synechocystis* sp. have a higher affinity for adsorbing Cd(ii) and Cr(vi), respectively, compared to other tested HMs. This observation aligns with the HM adsorption rates obtained from the ICP-MS results. These results indicate that the surface of the cyanobacterial cells plays a crucial role in both maintaining cell suspension stability and interacting with HM ions.

The HM removal mode was adsorption as the intracellular removal mechanism requires long-term cultivation in contaminated water. After 7 days of cultivation, only 7–15% of Cd(ii) was accumulated intracellularly by *Anabaena doliolum*.^52^ Moreover, the biotransformation mechanism of HMs by enzymatic activity inside the cyanobacterial cells can contribute to HM removal, which requires a transition of metallic charges.^53^ We also know that metal homeostasis is important, but how metals transport in cyanobacteria is still not fully clear. Cyanobacterial cells can uptake a trace amount of intracellular HMs to maintain the concentration below the toxic level.^40^ In biotransformation, chemical and structural differences of metals can create specific intracellular effects of protein denaturation and uncoupling of oxidative phosphorylation; thus, these oxidative state changes can limit the amount of HM adsorption by cyanobacteria, and finally, the intracellular concentration of the HMs can be observed after a long period of cultivation.^54^ These results all support our conclusion that after a short culture period (48 h) the mechanism of HM removal is mainly surface adsorption by different binding sites on the cell surface of these two cyanobacteria.

## 4. Conclusion

The empirically supported conclusions suggest that two scrutinized live cyanobacteria *Synechocystis* sp. and *A. variabilis* exhibit substantial potential, showcasing numerous prospective applications in the realm of cyanoremediation as they promote the reduction of the concentration of toxic metals such as As(v), Cd(ii), Cr(vi), and Pb(ii) from aqueous solution with almost 50% cell viability. This result demonstrates that cyanobacteria exhibit variable binding affinities toward distinct HM ions, allowing for their alternate association with these metal ions. This finding further elucidates that the main mechanism underlying HM removal involves surface adsorption of cyanobacteria. From the preceding discourse, we can deduce three main conclusions: firstly, *Synechocystis* sp. and *A. variabilis* can effectively reduce HM concentrations from solution. Secondly, numerous surface binding sites are responsible for HM binding onto the cell surface of experimental cyanobacteria, with the predominant adsorption mode being primarily extracellular adsorption. Thirdly, the cyanobacterial efficacy of HM removal is contingent upon the specific strain and dosage, exhibiting a trend where metal removal efficiency diminishes with escalating metal concentration. Furthermore, it can be concluded that *Synechocystis* sp. with an initial concentration of 2 mg L^−1^ can remove 91% of Cr(vi), while *A. variabilis* can remove a maximum of 78% of Cd(ii) at 1 mg L^−1^ initial concentration, which can be used as benchmark reference standards for future advanced research in the field of cyanoremediation. Finally, it is posited that this article will prove instrumental in influencing the future trajectory of economically sustainable and scientifically sound cyanoremediation methodologies, particularly emphasizing the recovery of HMs from wastewater through cyanobacterial interventions.

## Author contributions

Md. Sabbir Hossain: conceptualization, investigation, data curation, visualization, formal analysis, methodology, writing an original draft. Tatsufumi Okino: supervision, validation, writing-review & editing.

## Conflicts of interest

There are no conflicts to declare.

## Supplementary Material

RA-014-D4RA00409D-s001
