# Supplementary material for: Cyanoremediation of heavy metals (As(v), Cd(ii), Cr(vi), Pb(ii)) by live cyanobacteria (Anabaena variabilis, and Synechocystis sp.): an eco-sustainable technology
Source: RSC Adv. 2024 Apr 2;14(15):10452–63. doi: 10.1039/d4ra00409d (PMC10986677; doi:10.1039/d4ra00409d)
Supplement: RA-014-D4RA00409D-s001 [file RA-014-D4RA00409D-s001.pdf]

Electronic Supplementary Material (ESI) for RSC Advances.
This journal is © The Royal Society of Chemistry 2024

Supplementary materials for:

Cyanoremediation of Heavy Metals (As (V), Cd (II), Cr (VI), Pb (II)) by Live Cyanobacteria (*Anabaena variabilis*, and *Synechocystis* sp.): An Eco-Sustainable Technology

Hossain Md Sabbir, Tatsufumi Okino*

Email address of the corresponding author: **okino@ees.hokudai.ac.jp**

Supporting information:

- Tables S1-S3
- Figures: S1-S8

Table S1. Experimental design of this study.

Experiments	Experimental conditions	
Effects of HMs on cyanobacterial growth and pigment production	HMs concentration:	
	Synechocystis sp.<br>Cd: 1 mg L^{-1}<br>As: 1 mg L^{-1}<br>Pb: 4 mg L^{-1}<br>Cr: 2 mg L^{-1}	*Anabaena variabilis*<br>Cd: 1 mg L^{-1}<br>As: 4 mg L^{-1}<br>Pb: 1 mg L^{-1}<br>Cr: 1 mg L^{-1}
	pH: 7.5	pH: 8.0
	Culture time: 12 days	Culture time: 14 days
Analysis of surface morphology change	Scanning electron microscope (SEM),<br>Fourier transform infrared spectrometry (FT-IR): lyophilized cell sample.<br>HMs concentration:	
	Synechocystis sp.<br>Cd: 1 mg L^{-1}<br>As: 1 mg L^{-1}<br>Pb: 4 mg L^{-1}<br>Cr: 2 mg L^{-1}	*Anabaena variabilis*<br>Cd: 1 mg L^{-1}<br>As: 4 mg L^{-1}<br>Pb: 1 mg L^{-1}<br>Cr: 1 mg L^{-1}
	pH: 7.5	pH: 8.0

	Culture time: 48 h	Culture time: 48 h
Surface negativity change: Zeta potential	HMs concentration:	
	Synechocystis sp.<br>Cd: 1 mg L^{-1}<br>As: 1 mg L^{-1}<br>Pb: 4 mg L^{-1}<br>Cr: 2 mg L^{-1}	*Anabaena variabilis*<br>Cd: 1 mg L^{-1}<br>As: 4 mg L^{-1}<br>Pb: 1 mg L^{-1}<br>Cr: 1 mg L^{-1}
	pH: 7.5	pH: 8.0
	Culture time: 48 h	Culture time: 48 h

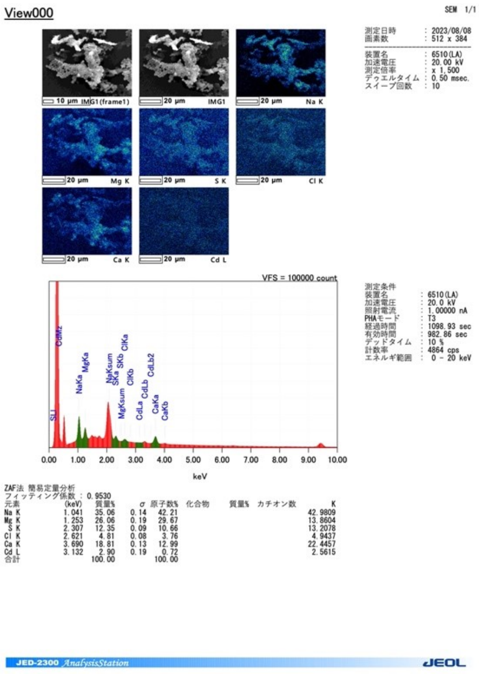

Figure S1. EDS mapping of *Synechocystis* sp. (NIES-3758) under Cd contamination.

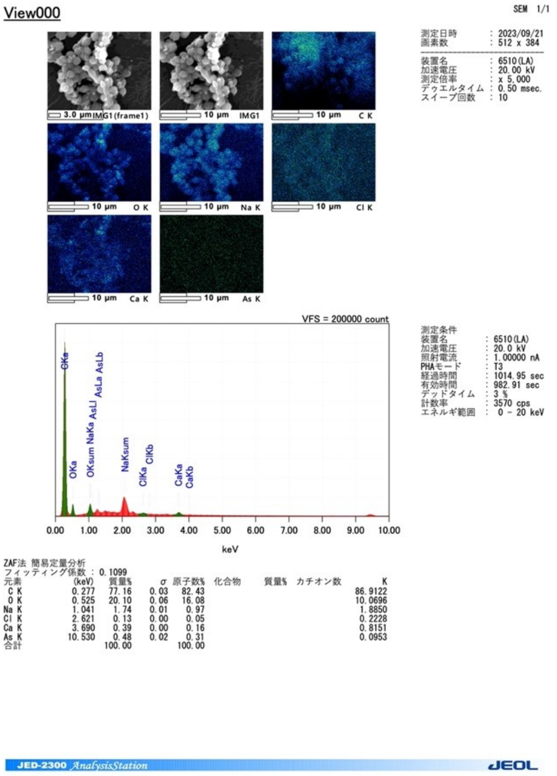

Figure S2. EDS mapping of *Synechocystis* sp. (NIES-3758) under As contamination.

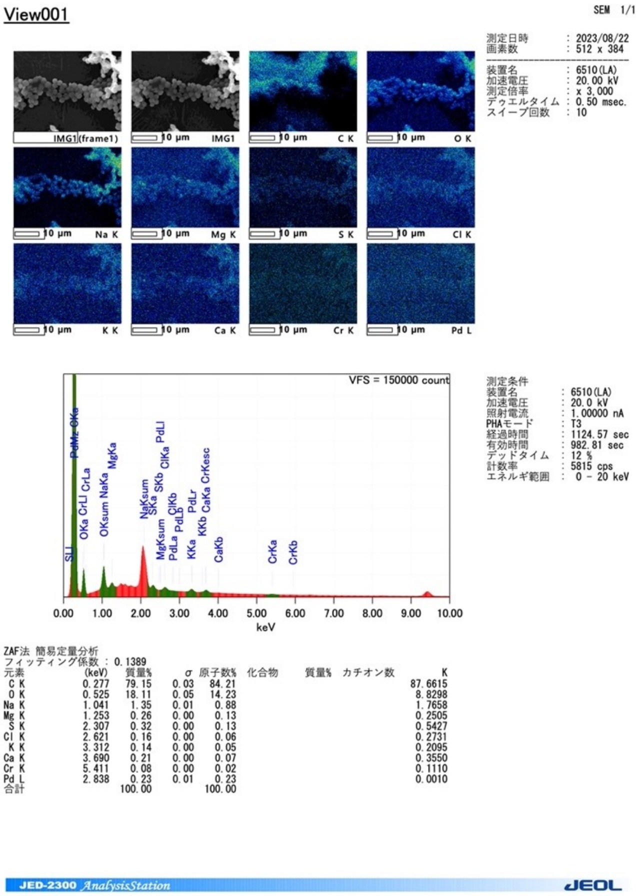

Figure S3. EDS mapping of *Synechocystis* sp. (NIES-3758) under Pb contamination.

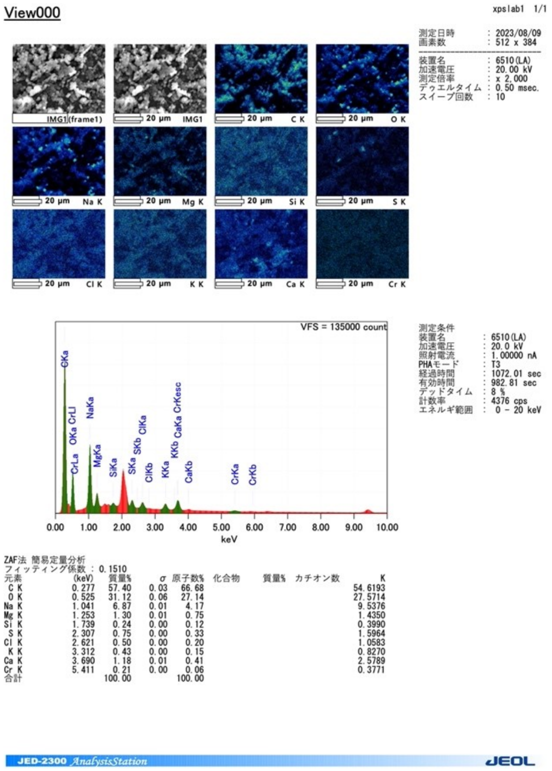

Figure S4. EDS mapping of *Synechocystis* sp. (NIES-3758) under Cr contamination.

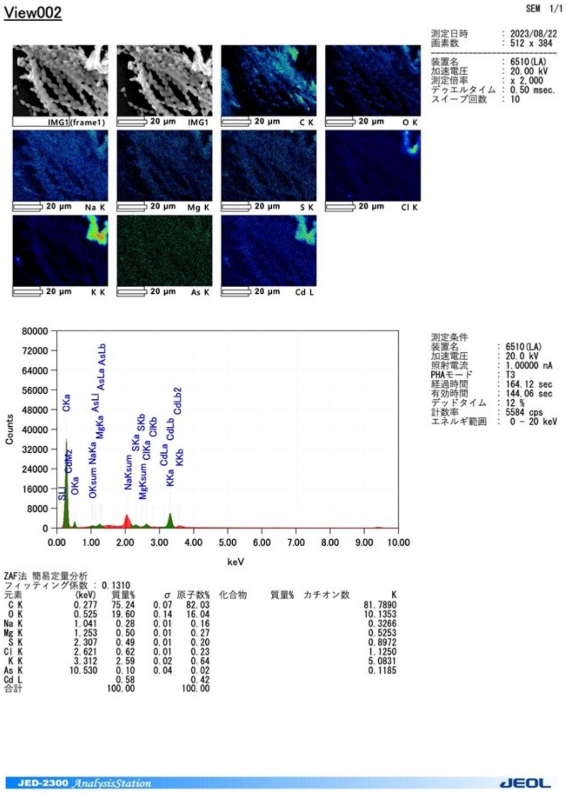

ZAF法 簡易定量分析
フィッティング係数 : 0.1310

元素	(keV)	質量%	σ	原子数%	化合物	質量%	カチオン数	K
C K	0.277	75.24	0.07	82.03				81.7890
O K	0.525	19.60	0.14	16.04				10.1353
Na K	1.041	0.28	0.01	0.16				0.3266
Mg K	1.253	0.50	0.01	0.27				0.5253
S K	2.307	0.49	0.01	0.20				0.8972
Cl K	2.621	0.62	0.01	0.23				1.1250
K K	3.312	2.59	0.02	0.64				5.0831
As K	10.530	0.10	0.04	0.02				0.1185
Cd L		0.58		0.42				
合計		100.00		100.00				

Figure S5. EDS mapping of *Anabaena variabilis* (NIES-2095) under Cd contamination.

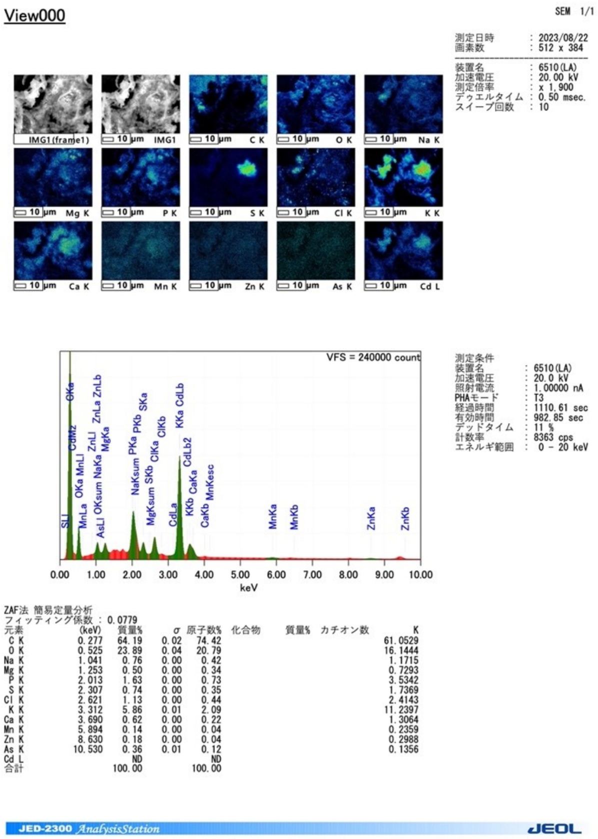

ZAF法 簡易定量分析
フィッティング係数 : 0.0779

元素	(keV)	質量%	σ	原子数%	化合物	質量%	カチオン数	K
C K	0.277	64.19	0.02	74.42				61.0529
O K	0.525	23.89	0.04	20.79				16.1444
Na K	1.041	0.76	0.00	0.42				1.1715
Mg K	1.253	0.50	0.00	0.34				0.7293
P K	2.013	1.63	0.00	0.73				3.5342
S K	2.307	0.74	0.00	0.35				1.7369
Cl K	2.621	1.13	0.00	0.44				2.4143
K K	3.312	5.86	0.01	2.09				11.2397
Ca K	3.690	0.62	0.00	0.22				1.3064
Mn K	5.894	0.14	0.00	0.04				0.2359
Zn K	8.630	0.18	0.00	0.04				0.2988
As K	10.530	0.36	0.01	0.12				0.1356
Cd L		ND		ND				
合計		100.00		100.00				

Figure S6. EDS mapping of *Anabaena variabilis* (NIES-2095) under As contamination.

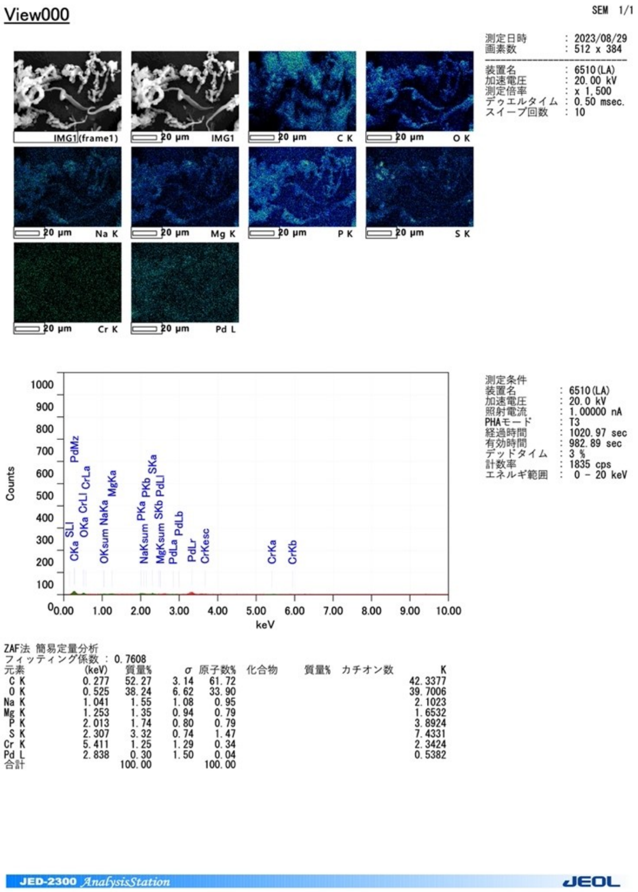

Figure S7. EDS mapping of *Anabaena variabilis* (NIES-2095) under Pb contamination.

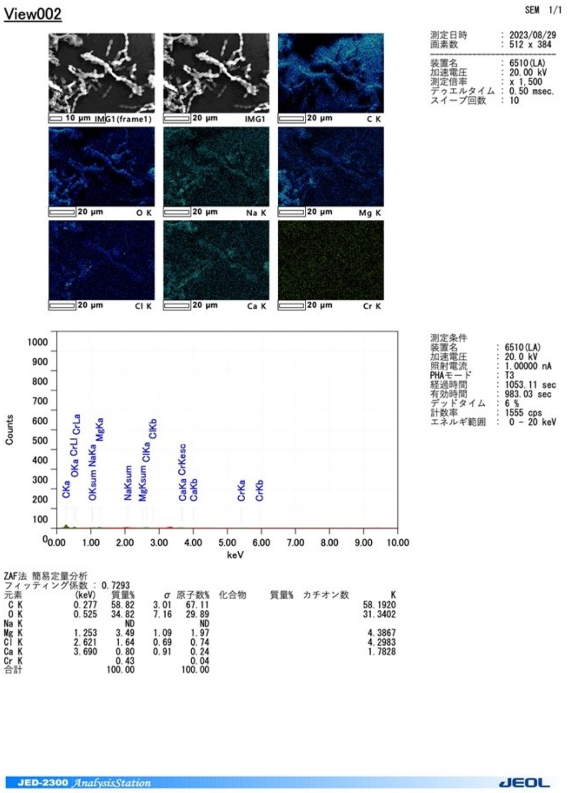

Figure S8. EDS mapping of *Anabaena variabilis* (NIES-2095) under Cr contamination.

Table S2. Zeta potential of *Anabaena variabilis*

Heavy metals	Excperiment-1 (mV)	Experiment-2 (mV)	Experiment-3 (mV)	Mean (mV)	Standard deviation
Control	-29.31	-29.51	-31.99	-30.27	1.49
Pb	-17.97	-19.22	-20.50	-19.23	1.27
As	-15.60	-15.90	-18.75	-16.75	1.74
Cr	-11.07	-16.55	-14.17	-13.93	2.75
Cd	-13.35	-11.99	-12.56	-12.63	0.68

Table S3. Zeta potential of *Synechocystis* sp.

Heavy metals	Excperiment-1 (mV)	Experiment-2 (mV)	Experiment-3 (mV)	Mean (mV)	Standard deviation
Control	-31.67	-28.79	-31.57	-31.57	1.63
Cr	-15.04	-16.91	-17.90	-16.91	1.45
As	-20.58	-18.03	-12.21	-18.03	4.29
Pb	-19.47	-22.30	-21.56	-21.56	1.47
Cd	-21.13	-24.35	-25.38	-24.35	2.21
